# Antiviral Activities of a Medicinal Plant Extract Against Sacbrood Virus in Honeybees

**DOI:** 10.1186/s12985-021-01550-y

**Published:** 2021-04-21

**Authors:** Liping Sun, Xueqi Zhang, Shufa Xu, Chunsheng Hou, Jin Xu, Dongxiang Zhao, Yanping Chen

**Affiliations:** 1 Institute of Apicultural Research, Chinese Academy of Agricultural Sciences, Beijing, 100093 People’s Republic of China; 2 Institute of Environment and Plant Protection, Chinese Academy of Tropical Agricultural Sciences, Haikou, 571101 Hainan People’s Republic of China; 3 USDA-ARS Bee Research Laboratory, Beltsville, MD 20705 USA; 4 Apiculture Institute of Jiangxi Province, Nanchang, 330052 People’s Republic of China; 5 Key Laboratory of Pollinating Insect Biology, Ministry of Agriculture, Beijing, 100093 People’s Republic of China

**Keywords:** *Apis cerana*, Chinese sacbrood virus, Herbal medicine, Antiviral agent, Immunity

## Abstract

**Background:**

Sacbrood is an infectious disease of the honey bee caused by *Scbrood virus* (SBV) which belongs to the family *Iflaviridae* and is especially lethal for Asian honeybee *Apis cerana*. Chinese Sacbrood virus (CSBV) is a geographic strain of SBV. Currently, there is a lack of an effective antiviral agent for controlling CSBV infection in honey bees.

**Methods:**

Here, we explored the antiviral effect of a Chinese medicinal herb *Radix isatidis* on CSBV infection in *A. cerana* by inoculating the 3rd instar larvae with purified CSBV and treating the infected bee larvae with *R. isatidis* extract at the same time. The growth, development, and survival of larvae between the control and treatment groups were compared. The CSBV copy number at the 4th instar, 5th instar, and 6th instar larvae was measured by the absolute quantification PCR method.

**Results:**

Bioassays revealed that *R. isatidis* extract significantly inhibited the replication of CSBV, mitigated the impacts of CSBV on larval growth and development, reduced the mortality of CSBV-infected *A. cerana* larvae, and modulated the expression of immune transcripts in infected bees.

**Conclusion:**

Although the mechanism underlying the inhibition of CSBV replication by the medicine plant will require further investigation, this study demonstrated the antiviral activity of *R. isatidis* extract and provides a potential strategy for controlling SBV infection in honey bees.

## Introduction

The Eastern honeybee (*Apis cerana*) is an important pollinator for crops and wild plants in Southeast Asia [31]. Compared to its close cousin European honeybee *Apis mellifera* which is the most widely managed crop pollinator worldwide, *A. cerana* has several advantages over *A. mellifera,* including the resistance to the parasitic mite *Varroa destructor* which is the most devastating pest of European honeybees, tolerance to low temperatures, and ability to utilize sporadic nectar sources in mountain and forest regions [14, 15, 30, 33]. However, the health of the Asian honeybee is seriously threatened by Sacbrood virus (SBV). SBV or *Morator aetatulas*, is an infectious virus belonging to the family of *Iflaviridae* and infects larvae of both European and Asian honeybees. The infected larvae fail to reach the pupal stage and die eventually. SBV was first detected in *A. mellifera* in the United States in 1913 [41] and has subsequently been reported in all major world regions where beekeeping practices are present [1, 9]. While SBV disease has been reported to affect about 15% of *A. mellifera* [27], it causes the most deadly and devastating disease in *A. cerana*. Historically, the catastrophic outbreak of SBV disease resulted in 95–100% mortality of *A. cerana* colonies in Thailand, Korea, China, and India [2, 11, 27, 32, 40].

SBV has evolved into multiple strains based on different geographical distribution. Chinese Sacbrood virus (CSBV) is a geographic strain of SBV infecting Chinese honeybee *A. cerana* [24]. CSBV primarily infects the 2nd to 3rd instar larvae of honeybees [23], resulting in failure to pupate and death, and eventually collapse of the whole colony [5]. CSBV was first found in *A. cerana* of Guangdong province in 1972 in China, and then spread rapidly to other regions of China and Southeast Asia and has been regarded as a major threat to *A. cerana* colonies [24].

So far, there still no effective treatment for CSBV infection. While CSBV infection can be partially relieved by replacing the queen or removing the infected combs from the beehives, such strategies are not effective ways to prevent further dissemination of CSBV among honeybees. RNAi has emerged as a potential method for combating viral diseases in honeybees [6]. Zhang et al. [44] reported that CSBV was significantly inhibited when honeybee larvae were fed with dsRNA corresponding to CSBV major capsid protein VP1 and RNAi-based treatment protected bee larvae from CSBV infection under laboratory conditions. However, the use of RNAi in honeybee disease control has been limited due to its high cost [39] and off-target effects [28], highlighting the need to develop new effective treatments for controlling CSBV infection in honeybees.

Over the years, natural products from plants that possess active ingredients and safety characteristics provide a rich source of candidate treatments for bee and hive health and show potential to be effective agents against bee pathogens, including viruses [29, 36]. Traditional Chinese herbal medicines display remarkable antiviral effects and have been widely used in the prevention and treatment of viral infectious diseases in humans and other animals [20]. We were motivated to explore the antiviral activity of a Chinese herbal medicine Radix isatidis (Banlangen and Daqingye in Chinese) for controlling CSBV infection in honeybees. R. isatidis is a commonly used traditional Chinese medicine famous for its broad-spectrum activity against various pathogens including human and avian influenza viruses [8, 43]. In this study, we provide evidence that R. isatidis extract could effectively inhibit the replication of CSBV in A. ceranae larvae, improve the immune response and extend the lifespan of CSBV infection larvae, clearly demonstrating an effective medicine for protecting honeybees from SBV infection.

## Materials and Methods

### Ethics Statement

Studies involved the Asian honeybee (*Apis cerana*), which is neither an endangered nor a protected species. Observations were made at the Institute of Apicultural Research, Chinese Academy of Agricultural Sciences (IAR-CAAS), Beijing, China. The apiary is the property of the IAR-CAAS and is not privately owned or protected in any way. No specific permits were required for the studies described.

### *Apis cerana* larvae samples

**Fig. 1:**
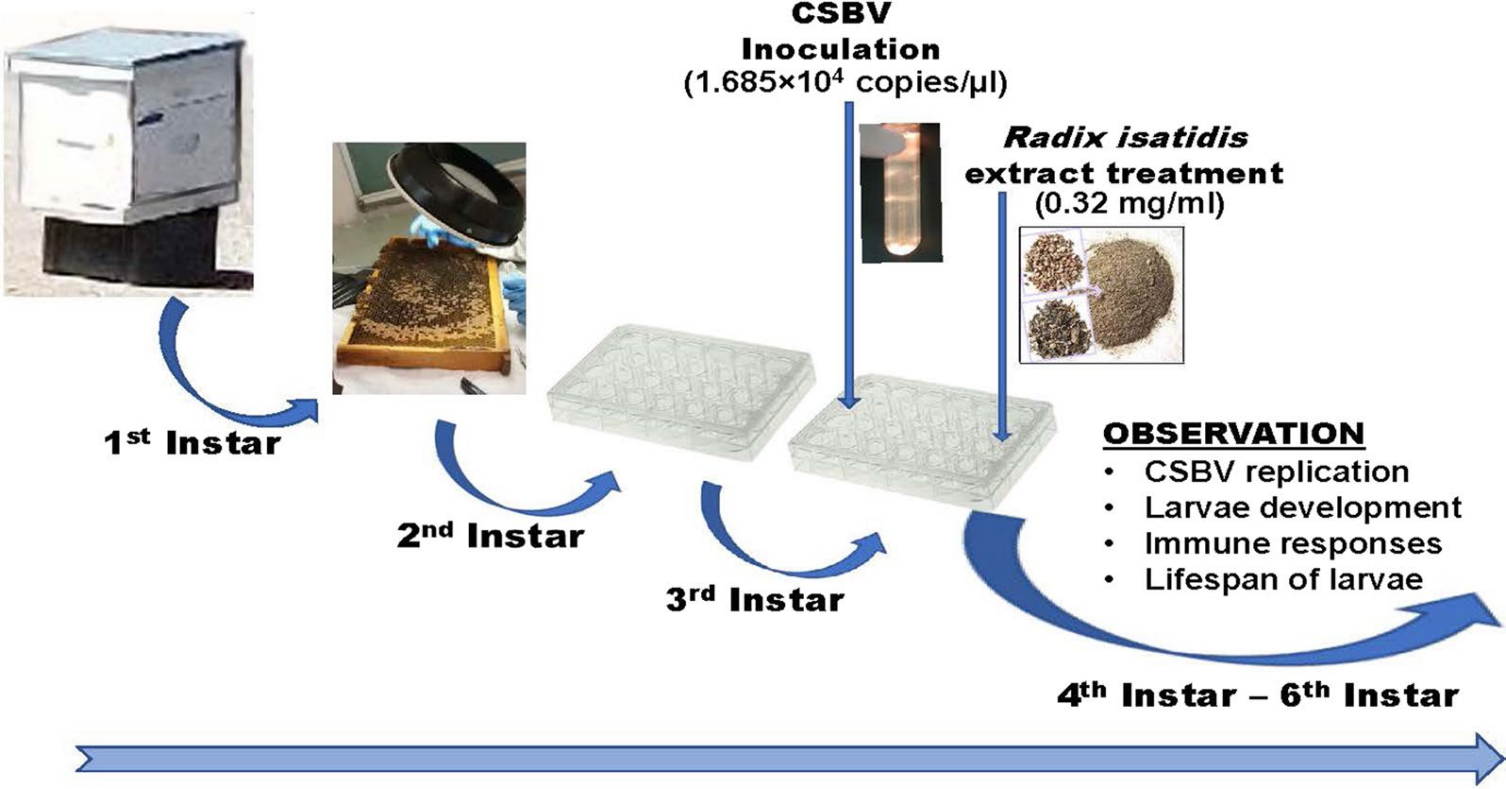
Graphic representation of the study design

Honeybee (*A. cerana*) colonies used in the study were originated from an experimental apiary maintained at the Institute of Apicultural Research, Chinese Academy of Agricultural Sciences, Beijing, China. In order to obtain the 2nd instar larvae, the queen from a health colony was restricted on a comb to lay eggs for 12 hours. After 48 hours, the comb with the 2nd instar larvae was taken out from the colony. The 2nd instar larvae were then transferred into 24-well plates individually. The 24-well plates were put into an incubator that was set at 32 ± 1 °C and 75 ± 5% relative humidity. The larvae were fed with man-made larval food and replaced with new diet each day. Detailed information on larval food used in the study is shown in Table 1. A diagram of the experimental design is shown in Fig. 1, and a thorough description of the experimental procedures is followed in the subsequent sections.

**Table 1:** Composition of food for different instar larvae (W/W %)

Instar larvae	Volume (µl)	Glucose %	Fructose %	Yeast %	Royal jelly %
2	20	6	6	1	50
3	20	7.5	7.5	1.5	50
4	30	9	9	2	50
5	50	9	9	2	50
6	80	9	9	2	50

### CSBV Purification

For purification of CSBV, infected larvae with significant disease symptoms were collected from field colonies. The presence of CSBV in infected larvae was confirmed by RT-PCR based on the description of Chen et al. [10]. CSBV-infected larvae (N = 200) were divided into two groups and homogenized in a 5 ml sterile phosphate buffer solution (PBS) separately with a sterile grinder. The homogenized mixture was centrifuged at 8000 rpm at 4 °C for 30 min. The supernatant was passed through a 0.20 μm cell filter to remove tissue debris and bacteria suspended in the solution. The collected CSBV solution was further purified through CsCl gradient centrifugations [19]. The CsCl was removed by dialysis against PBS, and CSBV purification was stored at −4 °C for the subsequent inoculation.

### RNA Extraction and PCR Amplification

Total RNA was extracted from CSBV infected larvae using an RNeasy Mini Kit (Adlai, Beijing, China) according to the manufacturer’s instructions. The cDNA was synthesized using a reverse transcription kit (Takara, Tokyo, Japan). PCR amplification was performed under the following conditions: initial denaturation at 94 °C for 5 min followed by 35 cycles of denaturation at 94 °C for 30 s, annealing at 58 °C for 30 s, extension at 72 °C for 15 s, and a final extension at 72 °C for 5 min. Then, a 593-bp fragment of the CSBV genome was amplified with primers described by Ma et al. [24]. The size of the PCR products was verified by electrophoresis on 1% agarose gel in 1 × TAE buffer. The primer specificity of the purified PCR products was confirmed by sequencing analysis.

In addition, PCR assays were performed for RNA extracted from CSBV infected larvae to exclude the presence of the other common bee viruses, including Acute bee paralysis virus (ABPV); Black queen cell virus (BQCV), Chronic bee paralysis virus (CBPV), Deformed wing virus (DWV), Israeli acute paralysis virus (IAPV), and Kashmir bee virus (KBV) following methods described in references [3, 4, 26, 34, 37, 38].

### Determination of CSBV Concentration

The concentration of CSBV purification described above was determined by absolute quantitative Polymerase Chain Reaction (qPCR) using the standard curve method. The forward and reverse primers (5′-ccttggagtttgctatttacg-3′ and 5′-cctacatccttgggtcag-3′) were used to amplify a 161 bp CSBV fragment. The qPCR was carried out in BIOER LineGene 9600 real-time PCR system [17, 18]. The qPCR reaction mixture contained a total of 15 μl reaction mixture with 0.3 μl each of forward and reverse primers, 7.5 μl SYBR, 1 μl cDNA template and 5.9 μl water. The PCR reaction began with a single cycle at 95 °C for 3 min followed by 40 cycles at 95 °C for 3 s, 60 °C for 1 min and 70 °C for 30 s. The amplified PCR products of CSBV were purified and inserted into a plasmid vector pMD-18 T (Takara, Japan) to generate recombinant plasmid DNA. A standard curve for a dilution series of recombinant CSBV plasmid DNA ranging from 100 to 109 genomic copies was established by plotting CT values vs. the log of the concentration of genome copies. The solution of CBSV purification was determined to contain 6.74 × 104 CSBV copies per microliter and defined as initial concentration.

The initial concentration of CSBV was diluted with larval diet at a ratio of 1:1, 1:3 and 1:9 corresponding to 3.37 × 104, 1.685 × 104, and 6.74 × 103 copies/µl, respectively.

### CSBV Inoculum

The 3rd instar (3-day-old) larvae that were reared in 24-well culture plates were divided into three groups by adding 5 µl of 3.37×10^4^, 1.685×10^4^ or 6.74×10^3^ CSBV copies/µl into 20 µl larval food (Table 1), respectively. At the same time, the larvae in the control group received the regular larval diet (Table1). Each group contained three 24-well culture places (N = 72 larvae). The plates were placed into an incubator (32 °C with humidity of 75%). According to the larval mortality results, group II inoculated with 1.685×10^4^ CSBV copies/µl larval diet resulted in close to 50% lethal rate (LD_50_) and therefore the viral concentration of 1.685×10^4^ CSBV copies/µl was selected for the subsequent evaluation of in vitro antiviral activity of *R. isatidis* extract against CSBV.

### *Radix isatidis* Extract Preparation

The roots and leaves of *Radix* *isatidis (I. indingtica Fort.*) were purchased from Beijing Hongda Kelai Biotechnology Co., Ltd. The equal quantity of extract powder of *R. isatidis* roots and leaves (1:1 ratio) was mixed in high-purity water at a concentration of 22.9 mg/ml, which was used as a stock solution. The stock solution was diluted with the larval diet (Table 1) into the final concentrations of 0.2 mg/ml, 0.32 mg/ml, and 0.43 mg/ml individually. Based on our pilot toxicity evaluation, 15 µl of *R. isatidis* extract at a concentration of 0.32 mg/ml was the most suitable dose to use for treatment as there was no significant difference in survivorship between the control group and the treatment group and was therefore chosen for the subsequent antiviral bioassays.

### Bioassay of the CSBV Inhibition with *R. isatidis* Extract

The 3rd instar (3-day-old) larvae that were reared in 24-well culture plates were divided into three groups: Group I (Negative control <NC> , fed with a regular diet without CSBV and *R. isatidis* extract); Group II (CSBV, inoculated with CSBV without *R. isatidi*s extract); and Group III- (CSBV&V *R. isatidis* extract, inoculated with CSBV and treated with *R. isatidis* extract). Each group contained three 24-well culture places (one plate was used for morphological study, one plate for assessing *R. isatidis* extract antiviral activity, and one plate for monitoring immune responses), making up biological replicates of twenty-four (N=24). In Group-II, each larva was fed with larval diet containing 5 µl of 1.685×10^4^ CSBV copies/µl larval diet while in Group-III, each larva was fed with diet containing both 5 µl of 1.685×10^4^ CSBV copies/µl larval diet and 15 µl of *R. isatidis* extract (0.32 mg/ml). The volume of the diet increased each day as larvae instar increased (Table 1) and the larval food was changed every day. For group II and III, the virus-containing food was replaced with regular larval food after 24 hours of inoculating with CSBV. For group III, the *R. isatidis* extract was provided to larvae from the 3^rd^ instar (3-day-old) to the 6^th^ instar (6-day-old). During the feeding process, the food was ensured to be kept on the bottom side of the culture plate to avoid contact with the larvae.

To evaluate the impact of *R. isatidis* extract on the development and survival of the CSBV infected larvae, the larval development in terms of morphology was observed under a stereomicroscope in a rapid manner to avoid the disturbance of the bright light to the developing larvae and recorded daily. The dead larvae were recorded and removed daily. The larval survivor rate between the 4th instar (4-day-old) and the 6th instar (6-day-old) among different groups was recorded and compared.

To assess the antiviral activity of R. isatidis extract against CSBV, five larvae were sampled daily for each group for three days post CSBV inoculation and R. isatidis extract treatment. The CSBV copy number at the 4th instar, 5th instar, and 6th instar larvae was measured by the absolute quantification PCR method as described above and compared among three different experimental groups.

To monitor immune responses of CSBV infected larvae during R. isatidis extract treatment, eight larvae were sampled daily for each group for three days post CSBV inoculation and R. isatidis extract treatment. The expression of four genes encoding antimicrobial peptides apidaecin, abaecin, hymenoptaecin and defensin at the 4th instar, 5th instar, and 6th instar larvae was measured and compared among three different experimental groups by relative quantification PCR method (2−ΔΔCt method) [35]. The primers of immune genes and housekeeping gene β-actin were described by Liu et al. [22] and Chaimanee et al. [7]. The PCR reaction was carried out using a BIOER LineGene 9600 real-time PCR system. The qPCR system contained a total of 20 μl with 0.8 μl each of primer, 10 μl SYBR, 1 μl cDNA template and 7.4 μl water. The PCR reaction began with a single cycle at 95℃ for 3 min, 35 cycles of 95℃ for 30 s, 60℃ for 30 s, 72℃ for 30 s. qPCR data analysis was followed. The qPCR data analysis followed the procedure described in Liu et al. [22].

### Statistical Analysis

The standard curve method was employed for the absolute quantification of CSBV. The relative expression level of the antibacterial peptide target gene was calculated by 2^−△△CT^ method. The results were expressed as mean ± standard deviation (SD). The one-way analysis of variance (ANOVA) and Tukey's Honestly Significant Difference (HSD) test were used to compare the difference in the copy number of CSBV, survivor rate, and abundance of immune transcripts among three different groups using SPSS 22.0 (SPSS, Chicago, Illinois, USA). Percentage data (survivor rate) were Arc Sine transformed before the statistical analysis. A *p*-value of ≤ 0.05 was regarded as statistically significant.

## Results

### *R. isatidis* extract could reverse the effects of CSBV on larval growth and development

**Fig. 2:**
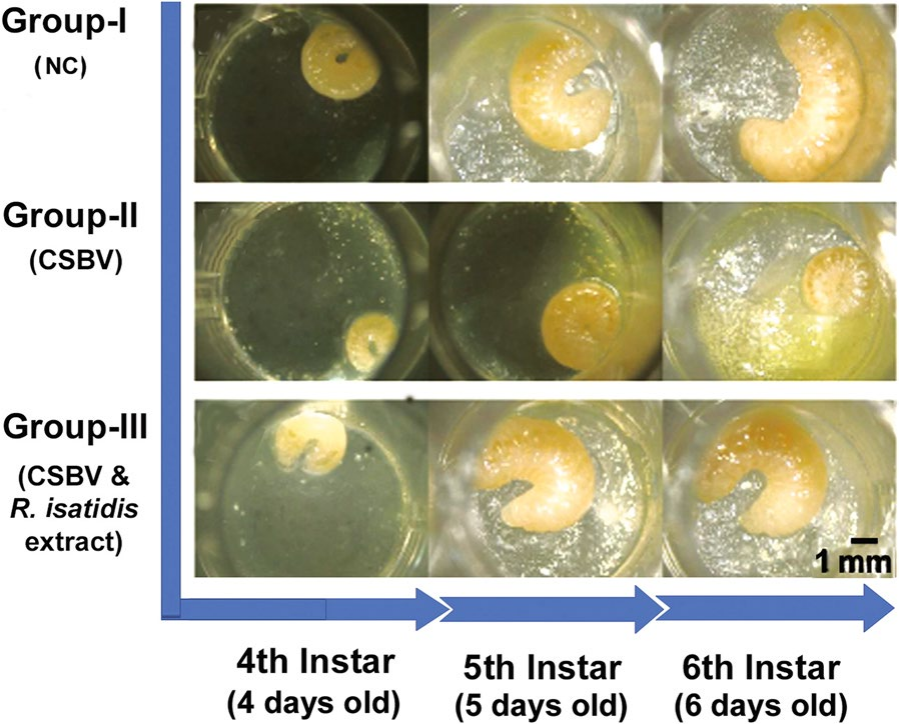
A representative figure showing the effect of *R. isatidis* extract for mitigating the impacts of CSBV on larval growth and development. In Group-I (CK), larvae received neither CSBV no *R. isatidis* extract. In Group-II, larvae were inoculated with CSBV without treatment of *R. isatidis* extract. In Group III, larvae were inoculated with CSBV and treated with *R. isatidis* extract. In Group-I and Group-III, the size of larvae increased significantly during each successive number of larval instars starting at the 4^th^ instar stage and ending at the 6^th^ instar stage. The CSBV infected larvae in Group-II showed impaired growth and development. Scale bar = 1 mm

The comparison of the morphology of larvae across three different groups showed that CSBV could severely impact the growth and development of *A. cerana* larvae and that *R. isatidis* extract was able to reverse the negative effects of CSBV on larval growth and development. Of 24 larvae in each experimental group, 100% of larvae in Group-I displayed normal development, 60.92% and 39.98% of larvae in Group-II were arrested at the fourth and fifth instar, respectively, without further development. Meanwhile, 86.15% of larvae in Group-II showed normal development and only 4.76% and 12.26% of larvae were arrested at the fourth and the fifth instar, respectively. A representative larvae morphological development is shown in Fig. 2. In Group-I, healthy larvae were pearly white and curved into a C-shape. The size of larvae increased significantly during each successive number of larval instars starting at the 4th instar larval stage and the body color of larvae turned into light yellow once they reached to the 6th instar. Compared to larvae in Group-I, CSBV infected larvae in Group-II showed a severe delay in development. The size of the CSBV infected larvae in Group-II was significantly smaller than that of larvae in Group-I. In addition, the color of CSBV infected larvae became dark brown, and more food is left on the bottom of the plate. Meanwhile, the CSBV infected larvae treated with *R. isatidis* extract in Group-III displayed a similar growth and development as larvae in Group-I. There was no significant difference in overall larval morphology and development between Group-I and Group-III (Fig. 2).

### *Radix isatidis* Extract Could Inhibit the Replication of CSBV

**Fig. 3:**
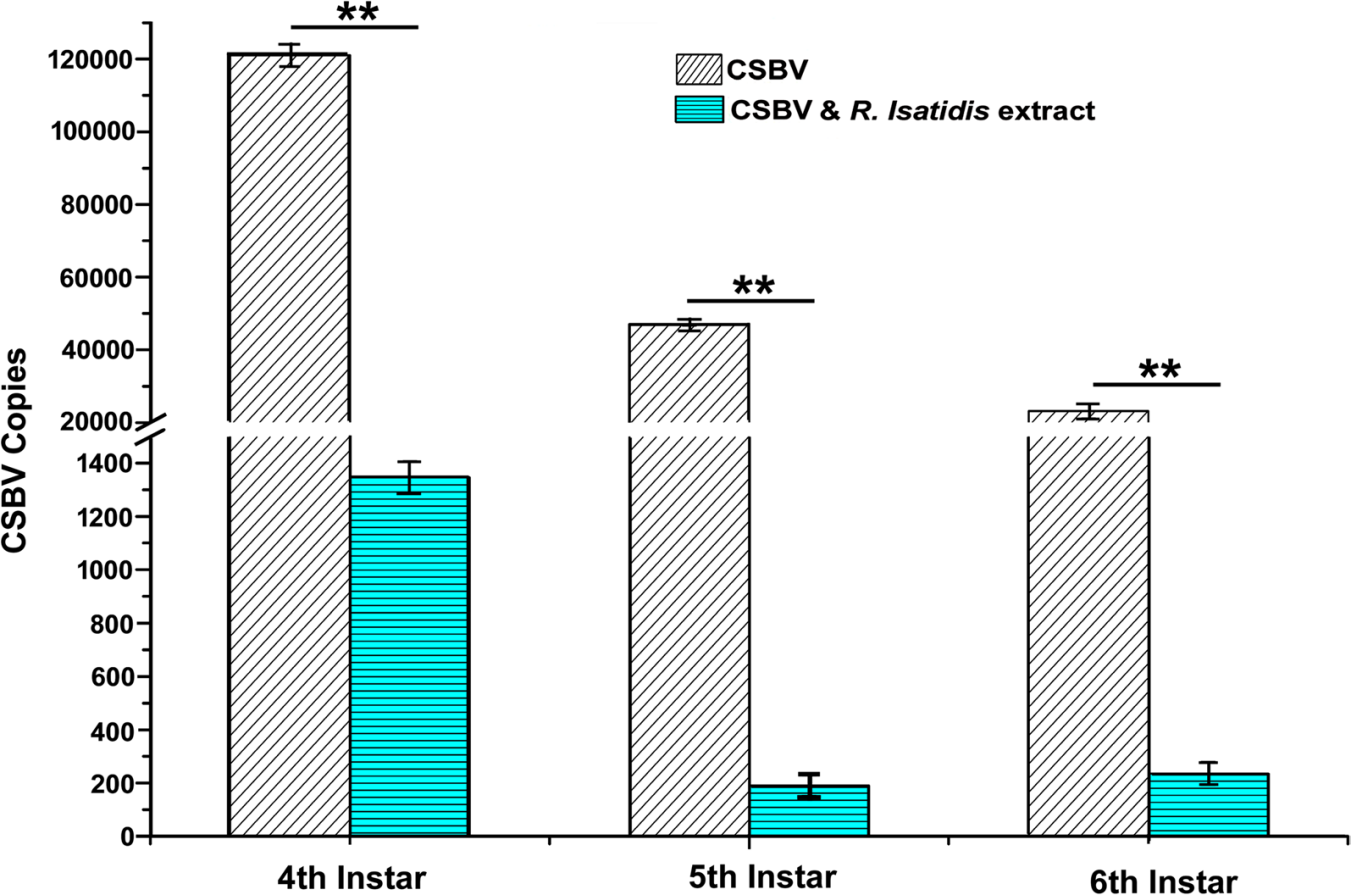
Inhibitory effects of *R. isatidis* extract on CSBV replication. In Group-II, larvae were inoculated with CSBV without treatment of *R. isatidis* extract. In Group III, larvae were inoculated with CSBV and treated with *R. isatidis* extract. Absolute RT‐qPCR measurement of CSBV gene copy number was conducted on the 4^th^ instar, 5^th^ instar, and 6^th^ instar larvae 24 h post CSBV inoculation for both Group-II and Group-III. Two asterisks (**) above denote a statistically significant difference between the two groups (*P* ≤ 0.01, Student’s t-test)

There was a statistically significant difference in the copy number of CSBV between Group II and Group III at different instar (4th instar: *p* < 0.01, 5th instar: *p* < 0.01, and 6th instar: *p* < 0.01, t-test). While there was no detectable level of CSBV in Group-I (N.C.), the copy number of CSBV in Group-II was 1.21 × 10^5^ copies/μl, 4.71 × 10^4^ copies/µl, and 2.328 × 10^4^ copies/µl in the 4-day-old, 5-day-old, and 6-day-old larvae, respectively. Compared to Group-II, a substantial decrease in CSBV copy number was observed in Group-III 24 h after *R. isatidis* extract treatment. The CSBV copy number in Group-III was found to continue to decrease steadily in response to the treatment of *R. isatidis* extract for 72 h. The CSBV copy number in Group-III larvae was 1.35 × 10^3^ copies/μl, 1.91 × 10^2^ copies/μl and 2.32 × 10^2^ copies/μl in the 4-day-old, 5-day-old, and 6-day-old larvae, respectively, clearly indicating the inhibitory activity of *R. isatidis* extract against CSBV in *vivo* (Fig. 3).

### *Radix isatidis* Extract Could Extend the Lifespan of CSBV-Infected Larvae

**Fig. 4:**
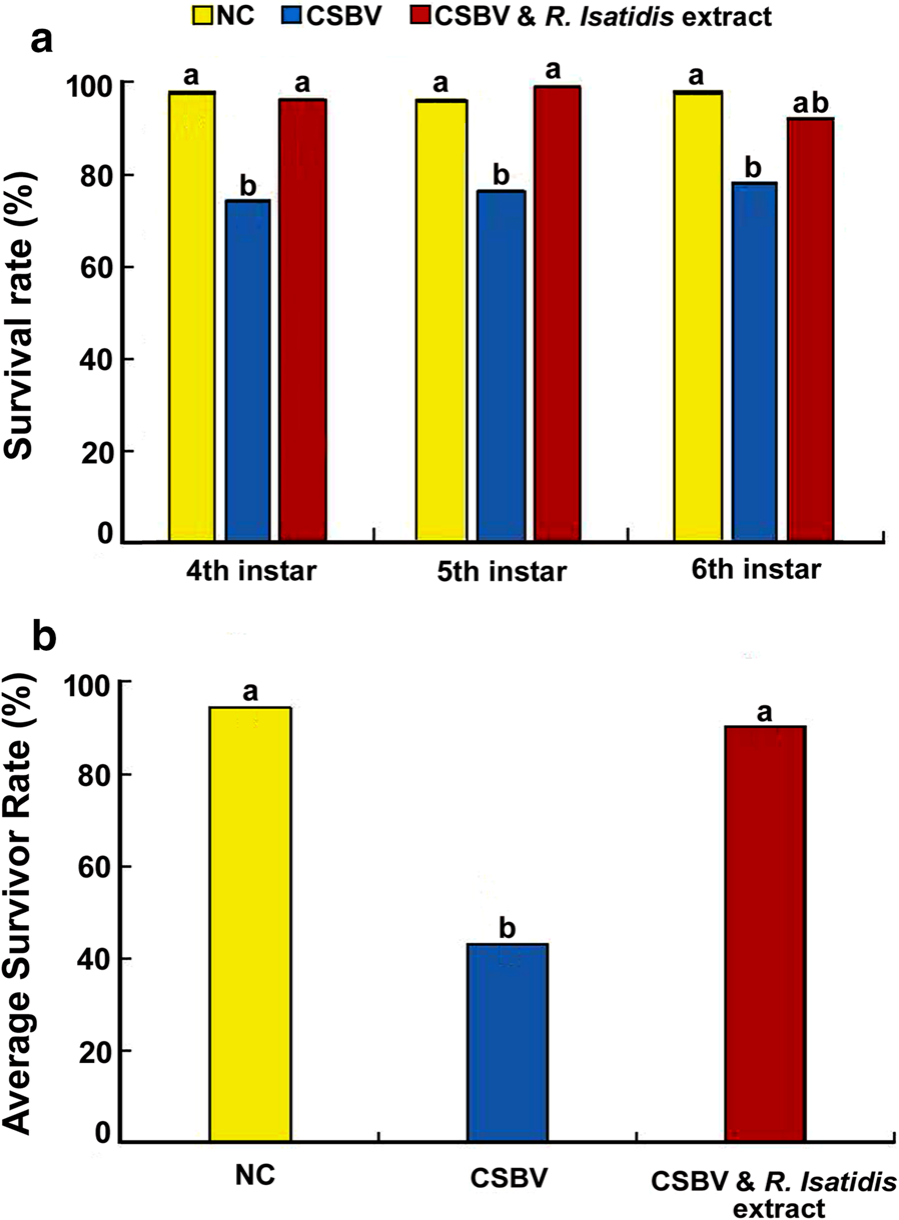
The effects of *R. isatidis* extract on extending the lifespan of CSBV-infected larvae. **a** The daily survival rate of different groups. **b** The overall survivor rate during a period of observation of different groups. In Group-I (CK), larvae received neither CSBV no *R. isatidis* extract. In Group-II, larvae were inoculated with CSBV without treatment of *R. isatidis* extract. In Group III, larvae were inoculated with CSBV and treated with *R. isatidis* extract. The different lower case letters above bars indicate the statistically significant difference among different groups (*P* ≤ 0.05, ANOVA and Tukey´s tests)

As shown in Fig. 4a, b, CSBV infection has a significant impact on larval survivorship. Larvae in Group-II displayed the highest mortality during the period of observation and at each instar stage among three groups. While the survival rate at Group-I was 98.61%, 97.16% and 98.61% for 4-day-old, 5-day-old, and 6-day-old larvae, respectively, the survival rate in Group-II was 75%, 77.3%, and 79.08% for 4-day-old, 5-day-old, and 6-day-old larvae, respectively. However, the survivorship of CSBV-infected bees was significantly improved by applying *R. isatidis* extract. The survival rate in Group-III was 97.22%, 100%, and 92.93% for 4-day-old, 5-day-old, and 6-day-old larvae, respectively. The overall survivorship during a period of observation was 98.61%, 43.05%, and 93.05% for Group-I, Group-II, and Group-III, respectively, clearly indicating that *R. isatidis* extract could result in a significantly improved survival of CSBV infected larvae.

One-way ANOVA and Tukey’s Honestly Significant Difference tests of the arcsine transformation of percentage data showed there was statistically significant difference in survivor rates among different experiment group. The survivor rate in Group II was statistically significantly lower than Group I. The *R. isatidis* extract treatment could improve the survivor of CSBV infected larvae as there was no statistically significant difference in survivor rate between the Group I and Group II at three different instar larvae (4th instar: *P* = 0.008, F(2,6) = 11.704, G-I vs. G-II *P* = 0.012, G-II vs. G-III *P* = 0.016, G-I vs. G-III *P* = 0.959; 5th instar: *P* = 0.001, F(2,6) = 25.144, G-I vs. G-II *P* = 0.006, G-II vs. G- III *P* = 0.001, G-I vs. G-III *P* = 0.210; 6th instar: *P* = 0.017, F(2,6) = 8.759, G-I vs. G-II *P* = 0.014, G-II vs. G-III *P* = 0.098, G-I vs. G-III *P* = 0.308; Average: *P* = 0.003, F(2,6) = 18.449, G-I vs. G-II *P* = 0.005, G-II vs. G-III *P* = 0.004, G-I vs. G-III *P* = 0.995)) (Fig. 4a, b).

### Relative Expression of Four Antimicrobial Peptides

**Fig. 5:**
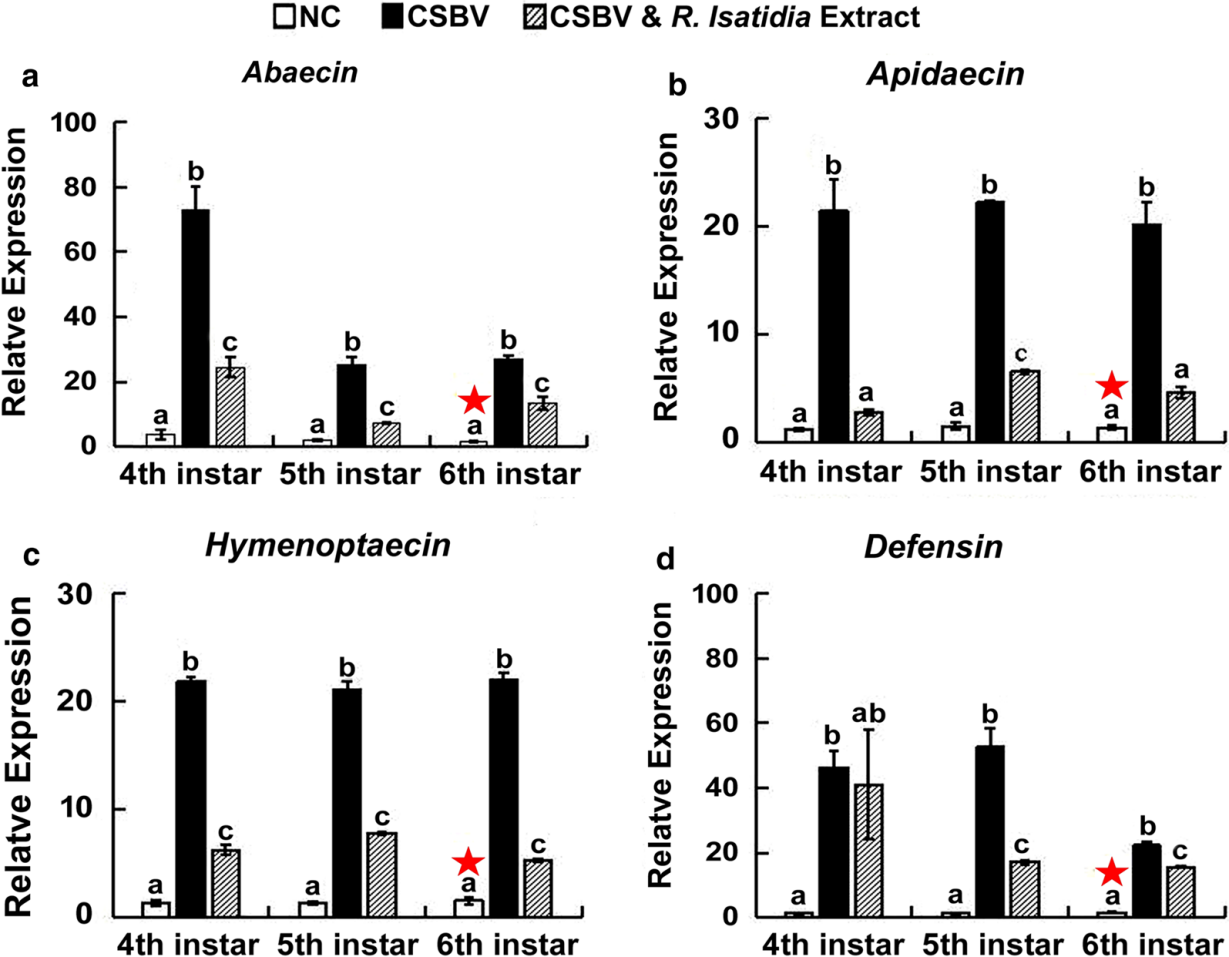
Relative changes of genes encoding antimicrobial peptides *abaecin* (**a**) *apidaecin* (**b**)*, hymenoptaecin* (**c**) *and defensin* (**d**). For each gene, the relative expression was expressed as an n-fold difference relative to the calibrator (marked by a star) by 2^–∆∆Ct^ method. In Group-I (CK), larvae received neither CSBV no *R. isatidis* extract. In Group-II, larvae were inoculated with CSBV without treatment of *R. isatidis* extract. In Group III, larvae were inoculated with CSBV and treated with *R. isatidis* extract. The different lower case letters above bars indicate the statistically significant difference among different groups (*P* ≤ 0.05, ANOVA and Tukey´s tests)

Relatve gene expression analysis showed that the expression levels of genes encoding antimicrobial peptides including *apidaecin*, *abaecin*, *hymenoptaecin* and *defensin* was activated in CSBV infected larvae (Group II) at different larval instar stages. One-way ANOVA and Tukey's Honestly Significant Difference tests showed the expression levels of four immune genes were significantly higher in Group-II than that in Group-I (*Abaecin—*4th instar: *P* = 0.000, F(2,6) = 62.091, G-I vs. G-II *P* = 0.0001, 5th instar: *P* = 0.000, F(2,7) = 104.196, G-I vs. G-II *P* = 0.0001, and 6th instar: *P* = 0.000, F(2,6) = 208.223, G-I vs. G-II *P* = 0.000; *Apidaecin—*4th instar: *P* = 0.000, F(2,6) = 43.127, G-I vs. G-II *P* = 0.0001, 5th instar: *P* = 0.000, F(2,6) = 1972.049, G-I vs. G-II P = 0.0001, 6th instar: *P* = 0.000, F(2,6) = 64.515, G-I vs. G-II *P* = 0.0001; *Hymenoptaecin*—4th instar: *P* = 0.000, F(2,6) = 709.958, G-I vs. G-II *P* = 0.0001, 5th instar: *P* = 0.0001, F(2,7) = 565.060, G-I vs. G-II *P* = 0.000, 6th instar: *P* = 0.000, F(2,6) = 687.439, G-I vs. G-II *P* = 0.0001; and *Defensin—*4th instar: *P* = 0.039, F(2,6) = 5.851, G-I vs. G-II *P* = 0.047, 5th instar: *P* = 0.000, F(2,6) = 56.606, G-I vs. G-II P = 0.000, 6th instar: *P* = 0.000, F(2,7) = 421.010, G-I vs. G-II *P* = 0.0001) (Fig. 5).

*R. isatidis* extract inhibited CSBV replication, which in turn led to the reduction in the intensity of the immune response in honey bee larvae. The relative expression levels of genes encoding *apidaecin*, *abaecin*, *hymenoptaecin* and *defensin* in Group III larvae was significantly lower than that in Group II, end an immune response. However, the immune response didn’t disappear completely after the treatment of *R. isatidis* extract as there was still significant difference in the relative expression levels of genes encoding *apidaecin, abaecin*, *hymenoptaecin* and *defensin* between Group I and Group III (*Abaecin*—4th instar: G-II vs. G-III *P* = 0.001, G-I vs. G-III *P* = 0.040, 5th instar: G-II vs. G-III *P* = 0.000, G-I vs. G-III *P* = 0.032, 6th instar: G-II vs. G-III* P* = 0.000, G-I vs. G-III *P* = 0.001; *Apidaecin*—4th instar: G-II vs. G-III *P* = 0.001, G-I vs. G-III *P* = 0.802, 5th instar: G-II vs. G-III *P* = 0.000, G-I vs. G- III *P* = 0.000, 6th instar: G-II vs. G-III *P* = 0.000, G-I vs. G-III *P* = 0.228; *Hymenoptaecin*—4th instar: G-II vs. G-III *P* = 0.0001, G-I vs. G-III *P* = 0.0001, 5th instar: G-II vs. G-III *P* = 0.0001, G-I vs. G-III *P* = 0.0001, 6th instar: G-II vs. G-I *P* = 0.0001, G-I vs. G-III *P* = 0.002; *Defensin*—4th instar: G-II vs. G-III *P* = 0.931, G-I vs. G-III *P* = 0.073, 5th instar: G-II vs. G-III *P* = 0.001, G-I vs. G-III *P* = 0.042, 6th instar: G-II vs. G-III *P* = 0.0001, G-I vs. G-III *P* = 0.0001) (Fig. 5).

Except for *defensin*, the other three immune genes had their peak of expression 24 h post CSBV infection and then declined thereafter. This inducible innate immune response to CSBV infection subsided with the treatment of *R. isatidis* extract. Except for the expression of *defensin* at the 4^th^ instar larvae, the expression levels of apidaecin, abaecin, and hymenoptaecin in Group-III expression were significantly lower than that in Group-II. The fold change in the gene expression levels between larvae in Group-II and larvae in Group-III was more than ten folds (Fig. 5).

## Discussion

Due to natural products' attractive properties such as safe, non-toxic, and biodegradable, they have been a rich source of medicines against various diseases including viral diseases. In this report, we provided evidence that the extract of a Chinese medicinal plant R. isatidis could inhibit honey bee SBV replication, modulate honey bees’ immune responses, and restore honey bees’ viability from SBV disease challenge, adding a new dimension to the role of the herb medicines in disease treatment and management.

Chinese sacbrood virus (CSBV) is the leading cause of A. cerana colony mortality, necessitating effective treatments that are safe, efficacious, and cost-effective. Herbal products have been used in traditional Chinese medicine for centuries. Previous studies have shown that Chinese herb medicines have unique roles in blocking viral replication or exerting direct or indirect antiviral effects [20]. R. isatidis (Ban-Lan-Gen) is a traditional Chinese herbal medicine that has been used for the prevention and treatment against a wide range of diseases, including viral diseases (reviewed in Zhou 2012). Several biologically active compounds have been isolated from R. isatidis and shown to have antioxidant and antiviral properties. For example, indirubin, a main active ingredient of R. isatidis, was reported to have potent antiviral and anti-inflammatory effects via inhibition of RANTES, is a member of a large family of cytokines that play a regulatory role in inflammatory processes [25, 27]. In addition, R. isatidis polysaccharides were found to inhibit the replication of human and avian influenza viruses [21]. Furthermore, Clemastanin B, and epigoitrin which are major phenylpropanoid compounds and abundant alkaloid in R. Isatidis, respectively, could effectively inhibit human and avian influenza viruses by blocking virus attachment and inhibiting virus multiplication [42, 43]. In our study, the dosage of 48 ug R. isatidis extract per larva each day (15 µL of 0.32 mg/mL R. isatidis extract) did not lead to toxic effects, indicating that R. isatidis is of safe and non-toxic for honey bees. Our results that CSBV load of the Group-III treated with R. isatidis extract was significantly lower than the virus control Group-II and that development and survival rate of Group-III was significantly higher than that of Group II demonstrated the significant antiviral activity of R. isatidis against lethal infections of CSBV. The results encourage future evaluation of R. isatidis extract as an antiviral agent for the treatment of other viruses in honey bees. Future studies are also needed to identify, isolate, and characterize specific active ingredients of R. isatidis that are responsible for inhibiting CSBV.

Innate immunity is the first line of defense against invading microorganisms in insects and consists of cellular and humoral responses [16]. Humoral response refers to the activation of downstream intracellular signaling molecules by germline-encoded pattern recognition receptors that recognize pathogen-associated molecular patterns and the production of soluble effector molecules, antimicrobial peptides (AMPs), in response to invaders. Several AMPs, including apidaecin, hymenoptera, abaecin and defensin which are regulated by two intracellular signaling pathways Toll and Imd/JNK have been described in the honey bee [12, 13]. During viral infection, the rapid production of AMPs as a part of the host defense response is necessary to promote virus clearance and to prevent virus spread within the host. Our study showed that CSBV infection induced the rapid elevation of expression levels of the AMPs apidaecin, hymenoptera, abaecin and defensin, reflecting that honey bee host’s innate immunity acted quickly to mount a first line of defense. The significant reduction in virus titer after the treatment with R. isatidis extract was accomplished with substantially subsided host immune responses as shown that the expression levels of four AMPs in Group-III expression were over ten-fold lower than that in Group-II. This result clearly demonstrated the immunomodulatory roles of the herbal extract. However, more research is needed to better understand the mechanism of *R. isatidis* in the protection against CSBV replication and the modulation of the innate immune response in the future.

## Conclusion

In conclusion, our findings clearly demonstrate that *R. isatidis* can be a significant antiviral therapeutic agent to inhibit CSBV infection in honey bees. The results obtained from this study may serve as a basis for further exploration of herbal medicinal plants or substances derived from them for the discovery and production of novel antiviral drugs for disease treatment in honey bees.

## Data Availability

All data supporting the conclusions of this article are included in this published article.

## Abbreviations

SBV: Sacbrood virus
CSBV: Chinese Sacbrood virus
IAR-CAAS: Institute of Apicultural Research, Chinese Academy of Agricultural Sciences
PBS: Phosphate buffer solution
ABPV: Acute bee paralysis virus
BQCV: Black queen cell virus
CBPV: Chronic bee paralysis virus
DWV: Deformed wing virus
IAPV: Israeli acute paralysis virus
KBV: Kashmir bee virus
qPCR: Quantitative Polymerase Chain Reaction
SD: Standard deviation
ANOVA: Analysis of variance
HSD: Honestly Significant Difference
NC: Negative control
AMPs: Antimicrobial peptides
